# Protein-assisted reductive nanofabrication: silk sericin as a capping-reducing agent in sustainable nanotechnology

**DOI:** 10.3762/bjnano.17.107

**Published:** 2026-11-06

**Authors:** Nipun Padha, Meenakshi Sharma

**Affiliations:** 1 Department of Zoology, Sri Sai University, Palampur [Himachal Pradesh], India

**Keywords:** bio-reduction, *Bombyx mori*, electron donor, metal nanoparticles, nanoparticle synthesis, sericin

## Abstract

Sericin is a water-soluble silk protein, which is currently discarded in silk processing and manufacturing as industrial waste during degumming raw silk. This review critically examines the molecular characteristics of sericin that may contribute to its reducing activity, its proposed roles in nanoparticle formation and stabilization, and the extent to which current mechanistic interpretations are supported by experimental evidence. The performance of sericin is further compared with other classes of bio-reductants, including plant polyphenols, microbial systems, free amino acids, and polysaccharides, with particular emphasis on reduction kinetics, stabilization, reproducibility, and practical applicability. Available evidence indicates that sericin can contribute to both metal-ion reduction and nanoparticle stabilization under appropriate aqueous conditions; however, the relative contributions of individual functional groups and the molecular basis of nanoparticle formation remain incompletely established. Reproducibility is also influenced by variation in sericin composition, molecular characteristics, and preparation conditions. In addition, challenges associated with mechanistic validation, scale-up, formulation stability, and long-term biomedical safety limit assessment of its broader technological applications. Sericin therefore represents a potentially useful biomaterial for sustainable nanofabrication, but further standardized characterization, quantitative mechanistic studies, and scalable process development are required to define its practical advantages more reliably.

## Introduction

Green nanotechnology has spent the last two decades searching for bio-reductant platforms that not only perform well in principle but are also practically applicable. Plant extracts act as powerful reductants, though the source and seasonal availability have been found to be a problem [1]. Microbial systems provide alternative attractive design solutions, though the rate at which the system operates has proven to be too slow for practical application. Silk sericin is a protein by-product of the silk degumming process. Recently, it has gained interest as a potentially valuable biomaterial [2] that can be utilised in the green formulation of nanoparticles; it is a biopolymer from unprocessed waste material and has the ability to act as a reductant and a stabilizer for nanoparticles [3].

The global production of silk fibers has been found to generate approximately 50,000–60,000 metric tons of sericin each year as waste material from the degumming process before textile processing [4]. Over the years, scientists have realised that this by-product, earlier considered as useless, has multiple properties that may be beneficial; one such property is the ability to act as a reducing agent in the production of nanoparticles. Thus, the valorization of sericin in green nanomaterial synthesis could minimize the need for synthetic reagents, which are generally used to produce the nanomaterials [5].

This review critically examines the structural characteristics of sericin, its proposed roles in nanoparticle reduction and stabilization, and the extent to which currently proposed mechanistic interpretations are supported by experimental evidence [6]. The review further compares sericin with other classes of bio-reductants, including plant polyphenols, microbial systems, free amino acids, and polysaccharides, with particular emphasis on reduction kinetics, stabilization, reproducibility, and practical applicability. Also, there is an evaluation of the problems that are actually faced during the production of nanoparticles.

## Review

### Silk sericin: structure, composition, and reductive capacity

#### Structure

Many consider sericin to be a one-molecule substance, but sericin is a complex mixture of different components. The cocoons of *Bombyx mori* have at least four major isoforms, namely Ser1, Ser2, Ser3, and Ser-p [7], with different molecular weights from 20 to over 400 kDa [8]. The distribution of sericin varies throughout the cocoon layers; sericin consists of various amino acids and different carbohydrate side chains. All isoforms are simultaneously extracted by the conventional alkaline degumming method, with the proportions depending on the silkworm strain [9] and time–temperature treatment. Such variability may contribute to differences in nanoparticle nucleation, growth, and final particle-size distribution when sericin is used for nanofabrication [10]. The amino-acid composition of sericin therefore represents an important variable in determining its interactions with metal ions and will define the starting point of the chemistry in the process of nanofabrication. The major constituents of sericin are serine (≈33 mol %), glycine (≈17 mol %), aspartate (≈16 mol %), glutamate (≈10 mol %), and threonine (≈9 mol %) [11]; a minor constituent is tyrosine (≈5 mol %), and the content may vary depending on the silkworm strain (Table 1).

**Table 1:** Major amino acid components of silk sericin.

Component	Approximate content	Reported functional role in nanoparticle synthesis	References

serine	≈33 mol %	provides hydroxy groups, has weak reducing ability at alkaline pH	[8,10–11]
glycine	≈17 mol %	primarily contributes to protein structure	[4,8,11]
aspartate	≈16 mol %	helps in metal ion coordination via carboxyl groups	[4,8,10]
glutamate	≈10 mol %	may contribute to metal-ion binding and surface stabilization	[8,11]
threonine	≈9 mol %	hydroxy groups participate in reduction at high pH	[8,10–11]
tyrosine	≈5 mol %	proposed major electron-donor role in metal-ion reduction	[8,11]

#### Roles of functional groups

**Figure 1:**
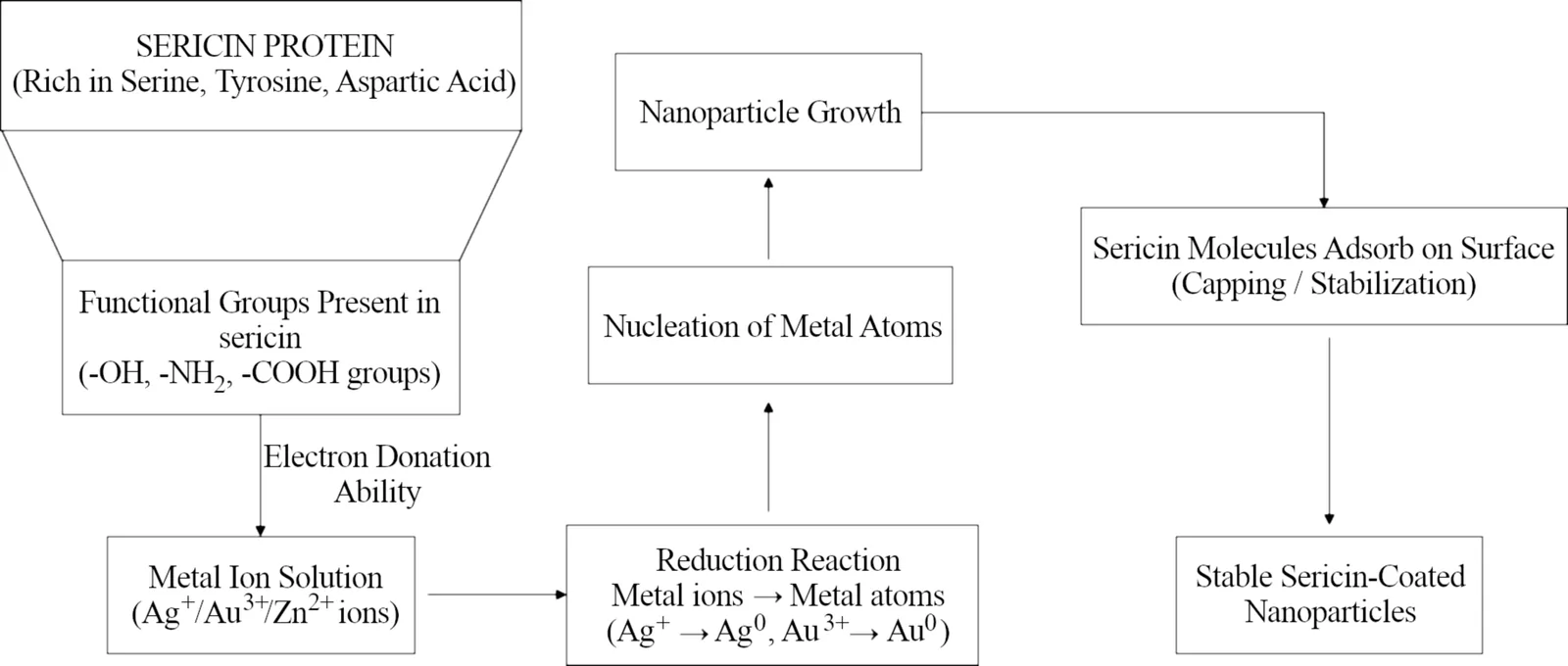
Mechanism of reduction of metal ions by sericin through electron donation.

The mechanistic assignment of tyrosine as the predominant electron donor (Figure 1) is the best-documented of those reported in the literature [10]. The phenolic hydroxy reduction potential of about +0.71 V vs the normal hydrogen electrode (NHE) at pH ≈7 is therefore suitable for reducing Ag^+^ (+0.80 V) and Au^3+^ (+1.0 V) [12]. In contrast, Zn^2+^/Zn has a much lower standard reduction potential (−0.76 V vs the standard hydrogen electrode [13]), indicating that Zn^2+^ is thermodynamically difficult to reduce to metallic Zn under mild biological conditions. Sericin can act as a stabilizing and capping agent in the production of zinc oxide nanoparticles [14]. Fourier transform infrared (FTIR) measurements, post-synthesis, universally show a reduction of phenolic O–H stretching at 3300 cm^−1^ and the appearance of bands of the carbonyls expected in quinone, the expected two-electron reduction product of phenol [15]. Competitive inhibition experiments using inhibitors such as tetranitromethane [16] and *N*-acetylimidazole [17], which are specific for the phenolic hydroxy group, confirm the inhibitory effects on the rate of synthesis. The role of the hydroxy groups present in the amino acids serine and threonine appears to be pH-dependent; their reducing activity is relatively weak under near-neutral conditions but increases under more alkaline conditions, particularly within the pH range of 9–11. Reactions performed at pH 10–11 produced smaller nanoparticles than reactions conducted near neutral pH. This behaviour may reflect increased participation of hydroxy residues under alkaline conditions; however, the effect of pH on sericin conformation, metal-ion speciation, and nucleation kinetics makes it difficult to attribute the observed size differences to a single molecular mechanism [4,10]. Thus, the relationship between pH and nanoparticle size should be interpreted as a combined chemical and conformational effect rather than as evidence for the isolated action of a single amino-acid residue. The carboxylates of aspartate and glutamate are mainly used to coordinate the metal ions and thus place them close to the reducing groups but do not directly reduce them [4]. This is in fact very important, because in some reports it is reported incorrectly thus leading to confusion between these two different roles, which will in fact hinder more than help in the mechanistic process.

Another underappreciated variable with an important role in nanoparticle synthesis, is the conformational state of sericin at the time of the introduction of the metal ion. Although the random coil conformation dominates in solution, detectable changes in the conformation favoring the formation of beta sheets are observed [8,18]. The solutions of sericin after an aging period of 48–72 h show higher levels of aggregation favoring the formation of beta sheets, giving rise to nanoparticles with higher polydispersity. The speed of temperature increase during the synthesis also played an important role, according to the same principle [10]. The rate at which the temperature is increased or decreased leads to the rise of different conformational landscapes, and thus to different size distributions. These are variables, but they have to be considered as variables from the start.

### Reduction mechanism

The sericin-mediated synthesis follows the Finke–Watzky kinetics model of a slow and continuous nucleation step followed by a fast and autocatalytic surface growth step [19–20]. The induction period, with no surface plasmon resonance absorption observed in the early stages of the ultraviolet–visible progress curves, is considered to be the most useful mechanistic parameter of the synthesis process [19]. It is the time where the number density of nanoparticles is being established; it is directly linked to the final size of the nanoparticles for a given metal precursor loading [21]. The induction period can be minimized by varying pH, temperature, and sericin concentration to produce more nanoparticles, smaller particles, and a narrower particle size distribution [5]. It is observed that sericin concentration has more impact on the rate constant of nucleation than on the rate constant of growth; hence, it acts as a nucleating agent rather than a reducing agent [20] (Table 2). A limitation of the two-step model is observed at higher sericin concentrations and temperatures, where the secondary nucleation during growth results in polydispersity; beyond this point, the model is not applicable [22].

**Table 2:** Parameters affecting sericin-mediated nanoparticle synthesis.

Parameter	Effect on nanoparticle formation	Mechanistic reason	References

pH	higher pH has been associated with smaller nanoparticles in some reported systems	changes in hydroxy-group activity, protein conformation, and metal-ion speciation may contribute	[4,10]
temperature	higher temperature can accelerate nanoparticle formation under reported conditions	increased nucleation and growth kinetics	[5]
sericin concentration	influences nucleation rate	higher sericin concentration has been associated with changes in nucleation behaviour under reported conditions	[5,20]
aging of sericin solution	increased polydispersity	β-sheet aggregation alters protein conformation	[8,18]
heating rate	changes particle size distribution	conformational changes during temperature ramping	[10]

The significance of the corona-stabilizing sericin layer around nanoparticles should be more appreciated than it is usually given. The electrophilic quinones resulting from tyrosine oxidation are capable of forming covalent adducts with nearby lysine side chains as well as with the metal surface [23]; thus, this corona consists of a complex of electrostatic and hydrogen bonding interactions and covalent bonding between anchoring groups and the surface. Competitive displacement experiments demonstrate that the sericin corona is unresponsive to displacement by bovine serum albumin or polyethylene glycol under conditions that are known to readily displace citrate or polyvinylpyrrolidone (PVP) coatings of comparable thickness [24], a distinction that is of practical importance to any biomedical application.

Post-synthesis FTIR analysis has frequently been used to identify changes in functional groups following sericin-mediated nanoparticle synthesis [10,25]. Such spectral changes can provide useful evidence for interactions between sericin and the nanoparticle surface or for chemical modification of specific functional groups. However, assignment of an FTIR peak shift to a specific mechanistic step requires caution because changes in protein conformation, metal coordination, oxidation state, and surface adsorption may occur concurrently. Representative studies that use FTIR data to assign specific mechanistic roles should therefore be interpreted together with complementary evidence rather than as standalone proof of a complete reaction pathway [25–26]. A more rigorous mechanistic framework will require time-resolved measurements and kinetic modelling capable of distinguishing nucleation, growth, reduction, and surface-stabilization processes [27].

### Sericin versus other bio-reductants

#### Plant polyphenols

Polyphenols such as gallic acid, tannic acid, and green tea catechins are among the most powerful reducing agents available for biological applications [28]. Their high density of hydroxy groups on the aromatics rings ensures rapid reduction of even Pt [29] and Pd [30] at room temperature and the generation of small, crystalline nanoparticles. For the applications where the most rapid and efficient reduction of nanoparticles with a separate added stabilizer is needed, polyphenols have been found to have a better reduction efficiency than sericin [31]. The primary disadvantages of polyphenols are related to stability and supply reliability. As they are small in size, polyphenols cannot provide macromolecular steric hindrance and are subject to nanoparticle aggregation in complex media. Oxidized polyphenols have pro-oxidant activity and could be subject to slow aggregation of nanoparticles when stored for long times [32]. A further limitation is that many studies using botanical extracts do not quantitatively characterize the concentrations of the active polyphenolic constituents, which can vary with plant species, geographical origin, season, and extraction method [33]. This variability can complicate the reproducibility and comparison of nanoparticle-synthesis results obtained using different botanical preparations.

#### Microbial systems

Microbial nanofabrication, specifically those utilizing dissimilatory metal-reducing bacteria and extracellular fungal nanofabrication, is arguably the mechanistically best understood class of bioreductive nanofabrication [34]. The depth of molecular-level understanding of the electron transfer pathways in *Shewanella oneidensis* alone sets a high bar for the sericin literature to aim for [35]. The practical limitations of microbial systems are real and underappreciated. Its biggest drawbacks are its 24–72 h synthesis time, contamination of the nanoparticle product with biological debris, sterility requirements that increase cost and complexity, and the difference between “scalable” as reported in the academic literature and scalable by anyone who has practically attempted to produce gram quantities per week [28,34,36]. Under the optimized conditions reported in the cited studies, sericin-mediated nanoparticle synthesis can be completed within approximately 2 h in aqueous media.

#### Free amino acids and polysaccharides

In case of free amino acids such as tyrosine, cysteine, and tryptophan there are very well-defined chemical reaction pathways that cannot be provided alone by sericin. Cysteine-mediated synthesis of Au nanoparticles has been demonstrated to produce well-defined Au–S bonds that are desired for subsequent bioconjugation [37]. The disadvantage is the subsequent stabilization process, where the free amino acids cannot provide any macromolecular steric stabilization, and aggregation happens readily in the absence of any stabilizer [38]. Thus, the presence of sericin may combine the reducing contribution of individual amino-acid residues with concurrent macromolecular stabilization, thereby providing a functional combination that is not readily reproduced by free amino acids alone [4,38]. Similarly, polysaccharides such as chitosan, starch, and hyaluronic acid have also been systematically mischaracterized in the synthesis of nanomaterials [39]. Under the aqueous conditions commonly used for low-temperature nanoparticle synthesis, polysaccharides such as chitosan, starch, and hyaluronic acid are generally considered to contribute more strongly to stabilization and surface interactions than to direct metal-ion reduction [39–40]. The presence of these compounds in the synthesis process has caused confusion that is harmful to the field of nanofabrication and the field of polysaccharide chemistry.

#### Sericin

When compared with its peers, sericin is not as effective as polyphenols, which have faster reduction kinetics [28,31], amino acids with better chemical specificity [37–38], polysaccharides possessing better bioconjugate specificity [39–40], and microbial systems, which are mechanistically better understood [34–35]. The potential advantage of sericin lies in its ability to combine reducing and stabilizing functions within a single protein-based material. In aqueous nanoparticle synthesis systems, where both functions are desirable, this combination may reduce the need for separate reducing and capping agents; however, the magnitude of this advantage depends on the sericin preparation, metal precursor, and reaction conditions [4,10,21]. This is, for most application scenarios, a more desirable set of properties than being better in one dimension but worse in another (Table 3).

**Table 3:** Comparison of major classes of bio-reductants. Comparisons are qualitative and refer to trends reported in representative nanoparticle-synthesis systems; performance depends on material, precursor, synthesis conditions, and preparation method.

Class	Reduction behaviour	Stabilization	Reproducibility	Major limitations	References

polyphenols	generally rapid in many reported systems	often limited	can be affected by source/composition	composition variability; aggregation/oxidation concerns	[28,31,33]
microbial systems	often slower than small-molecule reductants	variable	potentially high when biological conditions are controlled	long synthesis times; sterility and scale-up challenges	[34–35]
free amino acids	moderate/compound-dependent	limited	generally, chemically defined	often require additional stabilization	[37–38]
polysaccharides	usually limited direct reduction	often strong	depends on polymer/source	predominantly stabilizing role in many systems	[36,39]
sericin	moderate/condition-dependent	strong reported stabilizing role	affected by material heterogeneity	variability in composition due to different sources	[4,10]

### Applications

Reported applications of sericin-assisted nanoparticles include antimicrobial materials, biomedical formulations, catalysis, and environmental applications. Among these, sericin-containing silver nanoparticle systems have received particular attention in wound-care materials, including electrospun scaffolds and hydrogel-based formulations [41]. Antibacterial activity has also been reported for sericin-conjugated silver nanoparticles. It was also demonstrated that these nanoparticles inhibited the growth of *Escherichia coli* and *Staphylococcus aureus*, as well as *Klebsiella pneumoniae*, in vitro, supporting further investigation of sericin-based nanocomposites for antimicrobial applications [2,42]. Both electrospun scaffold and hydrogel dressing forms exhibit clear activity against the problem of biofilm formation, which is an issue in wound care. Sericin-stabilized silver nanoparticles are also less cytotoxic to mammalian keratinocytes than the equivalent citrate- or PVP-capped nanoparticles [10], which is due to the protein corona delaying the release of Ag^+^ [24]. This is one of the few applications that shows that the stabilizer choice makes a difference to the nanoparticles properties, a fact which is not necessarily true for all of the other applications discussed here. A sericin network surrounding palladium nanoparticles provides protection against the sintering of the particles under reaction conditions, which enable turnover numbers similar to the commercial Pd/C catalyst in the Suzuki–Miyaura reaction in aqueous media [43].

### Challenges

Reproducibility remains a major challenge because the molecular characteristics of sericin are often insufficiently characterized in nanoparticle-synthesis studies [4]. Parameters such as tyrosine content, molecular-weight distribution, and free amine concentration may influence synthesis outcomes [10]. Consequently, variability between laboratories may arise not only from differences in reaction protocols but also from differences in the composition and physicochemical properties of the sericin preparation itself. Establishing minimum reporting standards for sericin composition and molecular characteristics would therefore improve comparability between studies and help distinguish genuine process effects from reagent-related variability [10]. Until minimum standards are set for the characterization of sericin preparations, as with polymer chemistry standards for molecular weight and dispersity, reproducibility of results between labs will remain an issue, often ascribed to “protocol differences”, where the actual issue is with the reagents.

Mechanistic quantification is still far from complete, and a framework needs to be developed that connects sericin molecular parameters to nucleation and growth rate constants, and ultimately to nanoparticle properties. This will require the adoption of time-resolved spectroscopy and kinetic modeling [26], which are part of the physical chemistry toolbox, by the nanoparticle community, which has yet to happen, despite the ease with which the required instrumentation is available. Scaling up is not an afterthought following benchtop optimization, but a true engineering problem, which, in the case of inhomogeneous heat transfer, mixing, and nucleation gradients, results in a size distribution that is broader than that predicted from small scale data. Spray drying and freeze drying, which are required to obtain shelf-stable products, impose stresses on the sericin corona that solution stability [44], which is the most common approach to assess nanoparticle stability, does not predict. These issues require process engineering skills and industrial collaborations that are typically beyond the reach of independent academic groups. Finally, in vivo safety, which is relatively sparse, is in marked contrast to the biomedical applications frequently claimed in the nanoparticle sericin literature. Despite the fact that the biocompatibility of sericin is known, the long-term fate, that is, the stability of the corona following lysosomal enzymatic digestion, the immunogenicity of oxidized sericin, and the biodistribution implications of corona degradation, remains to be sufficiently characterized to allow for credible paths toward clinical translation.

## Conclusion

Silk sericin represents a promising protein-based component for green nanoparticle synthesis because it can contribute to both metal-ion reduction and nanoparticle stabilization under appropriate aqueous conditions. However, its performance should not be evaluated solely based on reduction efficiency. Compared with other bio-reductants, sericin offers a potentially useful combination of reducing, coordinating, and surface-stabilizing functions; but the relative contribution of these functions depends on the composition and conformation of the sericin preparation, the metal precursor, and the reaction conditions.

The available literature supports the use of sericin in several nanoparticle systems, including silver- and palladium-based materials, but the strength of evidence differs among applications. Antimicrobial and wound-care applications provide useful examples of how the properties of a sericin-containing surface layer can influence nanoparticle behaviour, whereas broader biomedical translation remains limited by incomplete information on long-term stability, degradation, immunogenicity, and biodistribution.

Several important limitations therefore remain unresolved. First, sericin preparations are heterogeneous and are not consistently characterized in sufficient molecular detail, which can contribute to variability between studies. Second, the mechanistic basis of sericin-mediated nanoparticle formation remains incompletely established, and post-synthesis characterization alone is insufficient to define a complete reaction pathway. Time-resolved spectroscopy, quantitative kinetic analysis, and better control of sericin molecular parameters are needed to establish clearer relationships between sericin composition, nucleation and growth processes, and final nanoparticle properties.

Finally, translation from laboratory-scale synthesis to reproducible and scalable production requires attention to mixing, heat transfer, formulation stability, storage, and process control. The current evidence therefore supports sericin as a potentially useful bio-based material for nanofabrication, while also indicating that standardized characterization, mechanistic validation, and scale-up studies are necessary before its broader technological and biomedical potential can be assessed with confidence. Further progress will depend on establishing reproducible material specifications, experimentally validated mechanistic models, and scalable synthesis strategies that enable meaningful comparison across laboratories and applications.

## Data Availability

Data sharing is not applicable as no new data was generated or analyzed in this study.
